# A Rare and Challenging Presentation of Empyema Necessitans/Necessitasis Leading to Brachial Plexopathy

**DOI:** 10.7759/cureus.8267

**Published:** 2020-05-24

**Authors:** Mohamad Abufaied, Phool Iqbal, Mohamed A Yassin

**Affiliations:** 1 Internal Medicine, Hamad Medical Corporation, Doha, QAT; 2 Internal Medicine, Hamad General Hospital, Doha, QAT; 3 Hematology and Oncology, Hamad General Hospital, Doha, QAT

**Keywords:** empyema, chest wall, brachial plexopathy, empyema necessitasis, empyema necessitans

## Abstract

Empyema necessitasis (EN) also referred to as empyema necessitans (EN) is a rare complication of empyema that can involve soft tissues outside the pleural cavity and can lead to brachial plexus injury. Although, brachial plexus injury most commonly occurs as a result of trauma, inflammation, or malignancies, but it has been rarely seen in EN.

We are reporting a rare and challenging case of empyema necessitasis (EN) causing impingement of brachial plexus in a 42-year-old, type 2 diabetic patient, who initially presented to the hospital with left-sided pleuritic chest pain and acute onset of left upper limb weakness. Urgent CT brain ruled out an acute neurological insult. Chest radiograph and contrast-enhanced CT thorax revealed left-sided loculated effusion. MRI of the left upper limb showed impingement of brachial plexus by the inflammatory process of surrounding effusion. Ultrasound-guided aspiration of the encapsulated fluid was performed and culture analysis of the fluid was remarkable for Prevotella oris (sensitive strain) growth. After drainage of the infected fluid and with a prolonged course of antibiotics, the patient's neurological symptoms improved significantly. Hospital course was uncomplicated and further follow-up was unremarkable for any deterioration.

Our objective is to emphasize on the prompt management with close follow-up in EN, which can present with life-threatening complications as seen in our case. Any delay can compromise the patient’s health. As per our limited knowledge, this case has not been reported in the literature.

## Introduction

Empyema is defined as pus in the pleural space from a complicated chest infection or pneumonia [1]. Pneumonia can lead to serious complications like para-pneumonic effusions, empyema, and life-threatening sepsis if associated with underlying immunosuppressive medical conditions, e.g., diabetes mellitus, lung cancer, alcoholism, and pulmonary aspiration [2-4]. About one-third of cases of empyema have no underlying etiology in the literature [4]. However, empyema fluid analysis has shown anaerobic bacterial growth of 36 to 76 percent with Prevotella species as the predominant one [5]. Empyema necessitasis also known as empyema necessitans (EN) can result as a rare complication of untreated empyema which tends to involve soft tissues of the chest wall outside the pleural cavity [6]. However, brachial plexus injury, which commonly occurs due to trauma, malignancy, and inflammatory processes, has not been reported much in the literature due to underlying EN [7].

## Case presentation

A 42-year-old gentleman, known case of uncontrolled type 2 diabetes mellitus, presented to the emergency room with two days history of left-sided chest pain and left upper limb weakness. Chest pain was gradual in onset, pleuritic in nature, and aggravated with deep inspiration. It was associated with a productive cough of yellow-colored sputum and minimal shortness of breath. There was no hemoptysis or foul-smelling sputum production. Left upper limb weakness started over two days and was acute in onset with no preceding trauma, dizziness, loss of consciousness, history of seizures, or any other neurological deficit. There were painful limb movements both actively and passively. The patient did not report any red flag signs like fever, night sweats, weight loss, or generalized body weakness. There was no history of intravenous drug abuse or contact with tuberculosis patients. His cardiac status was normal. Family history was unremarkable for any systemic disease like autoimmune diseases, tuberculosis infection, or malignancy. He has been on oral anti-diabetic medications but without regular monitoring or outpatient follow-ups.

Three weeks back, he was admitted to the hospital for empyema and underwent drainage of the infected fluid by chest tube thoracostomy with a video-assisted thoracoscopic surgery (VATS). ESBL (Extended Spectrum Beta-Lactamase) Escherichia coli (E. coli) bacterial organism was isolated from the fluid culture and treated with IV Ertapenem antibiotic for 14 days. The tissue histopathological report was unremarkable for any chronic granulomatous or malignant etiology.

On clinical examination, his heart rate, blood pressure, and respiratory rate were normal with the maintenance of normal room air oxygen saturation. Respiratory examination revealed left-sided stony dull percussion note and marked decreased breath sounds on auscultation, which were suggestive of left-sided pleural effusion. Posteriorly, the chest wall was asymmetrical with prominent non-tender swelling on the left side near the posterior axillary fold and unequal chest expansion, respectively. It was of normal skin temperature with no surrounding erythema, rash, or ulcer.

Left upper limb examination revealed reduced power of 2/5 and restricted active and passive movements mainly due to pain in the shoulder joint, while the rest of the respective joints i.e. elbow and wrists joints were normal. He was unable to abduct and flex his shoulder joint but adduction and extension movements were preserved. Sensations of the respective limb were intact. The rest of the neurological examination was normal. He was conscious, oriented in time and space, and walking without any neurological deficit.

**Figure 1:**
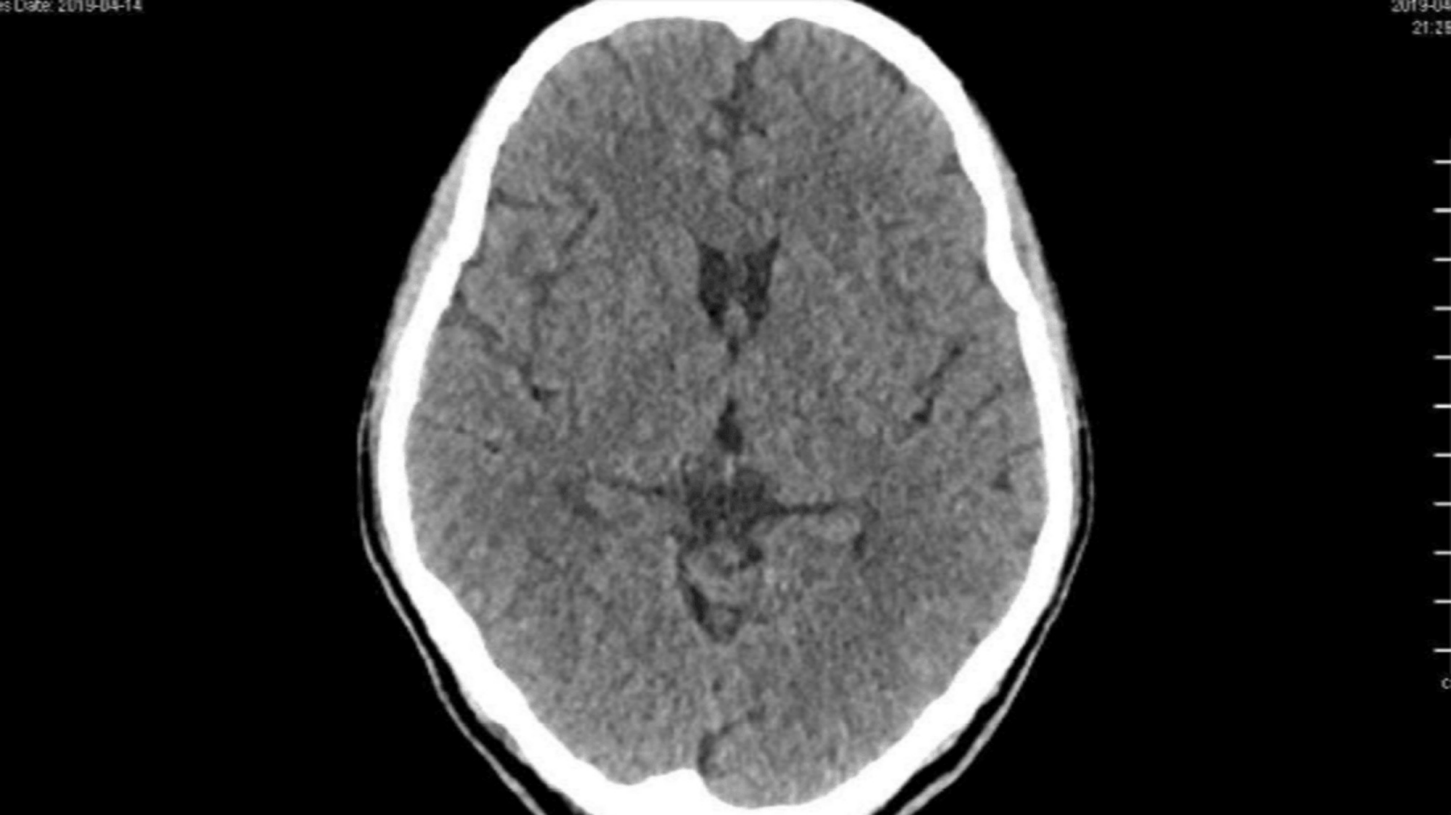
Normal CT head

**Figure 2:**
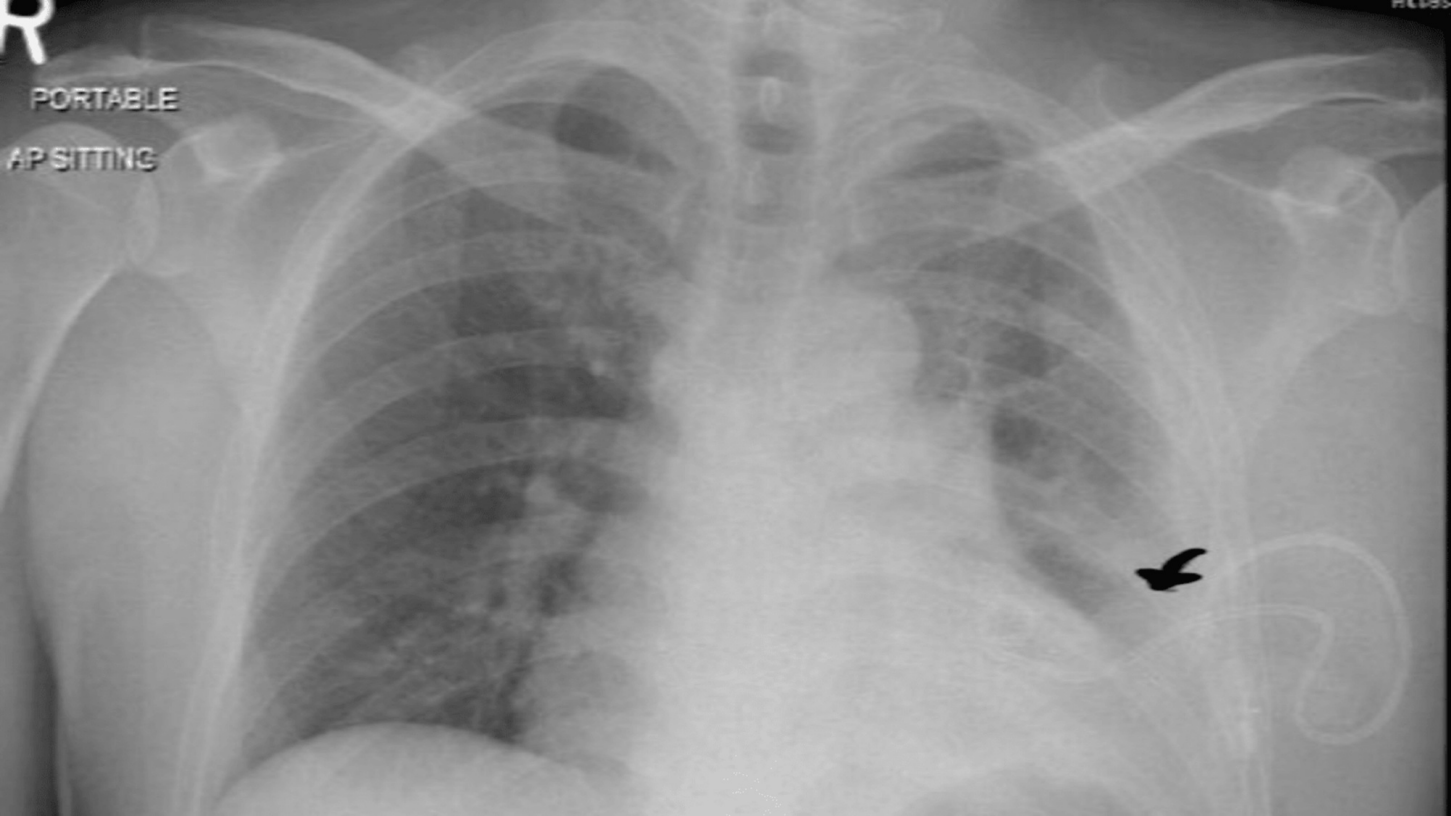
Chest X-ray showing left-sided pleural effusion

**Figure 3:**
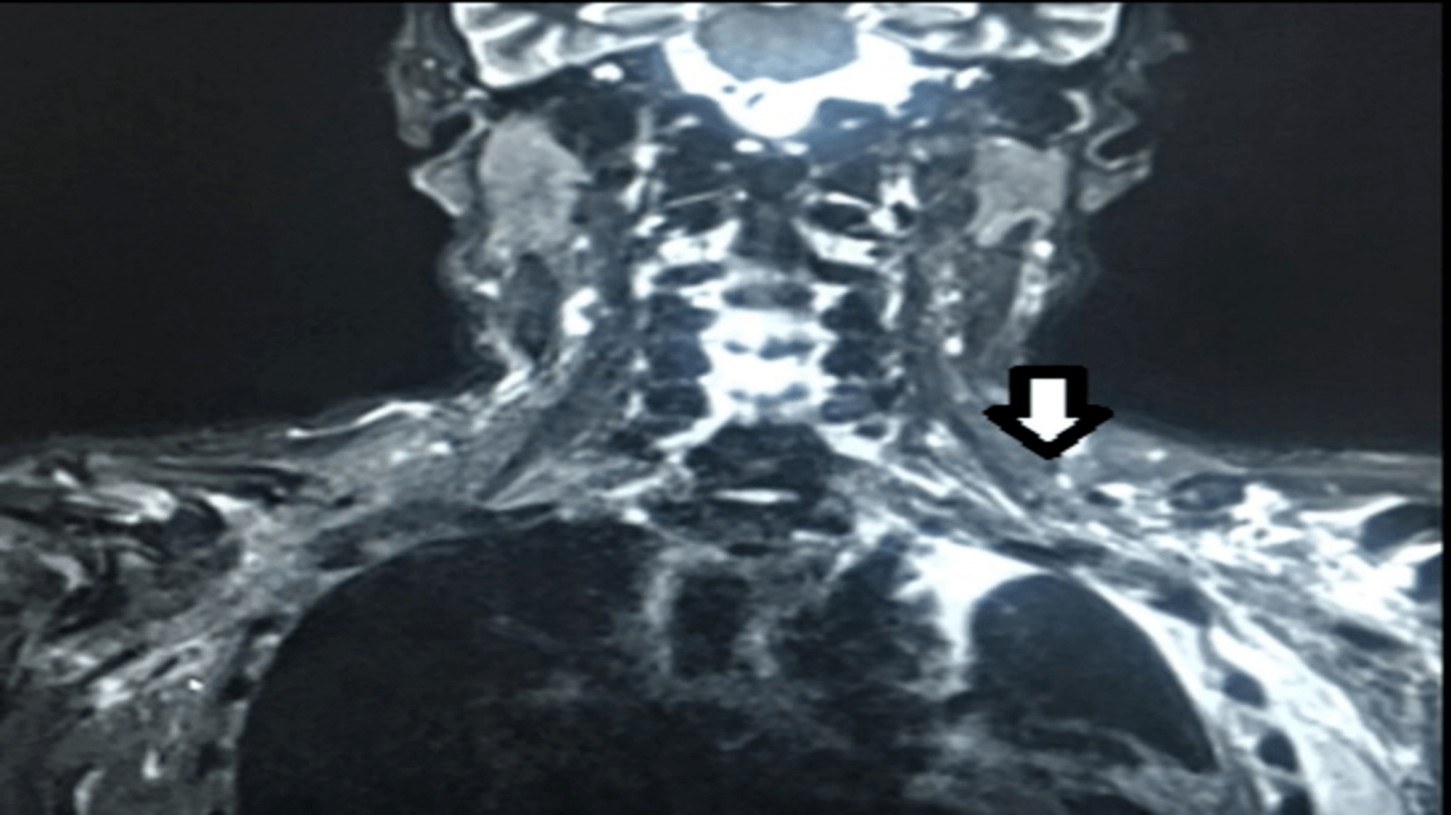
T2-weighted coronal view showing inflammatory process invading chest wall and surrounding left brachial plexus cords in left infra-scapular and axillary region

Urgent CT scan of the brain ruled out any acute insult as mentioned in Figure 1, while chest X-ray was remarkable for left-sided pleural effusion as in Figure 2. Further blood investigations showed neutrophilic predominant leukocytosis with increased inflammatory markers as shown in Table 1. However, his septic workup with aerobic and anaerobic blood cultures, respiratory viruses polymerase chain reaction (PCR) analysis, TB workup with an analysis of sputum smear for acid-fast bacilli (AFB) were negative. The patient was started empirically on IV Ertapenem antibiotic based on past infection history. Meanwhile, to investigate the etiology of his left arm weakness and pain, MRI of the left arm was performed which revealed loculated effusion involving the brachial plexus. The left brachial plexus was invaded by inflammatory process from underlying pleural effusion as shown in Figure 3.

**Table 1:** Inflammatory markers suggestive of ongoing infection

Blood investigations	Value	Normal range
White blood cells (WBC)	18.5 x 10^3^/uL	4.0-10.0^3^/uL
C-reactive protein (CRP)	399 mg/L	0.0-5.0 mg/L
Absolute neutrophil counts	16.5 x 10^3^/uL	2-7 x 10^3^/uL
Lactate	2.6 mmol/L	0.5-2.2 mmol/L

He was not improving on IV antibiotics. Clinically, there was persistent pleuritic chest pain and his inflammatory markers were also raised. It was decided to proceed with careful ultrasound (US) guided aspiration of the loculated effusion with the help of interventional radiologists as it was involving the brachial nerve plexus. After cautious fluid aspiration, the analysis revealed empyema due to Prevotella oris sensitive strain to Ertapenem antibiotic. The patient improved drastically in terms of pain and neurological deficit after fluid aspiration. He received a total of 28 days of antibiotics including 14 days of IV Ertapenem and later 14 days of oral ciprofloxacin 500 mg two times daily and Amoxicillin/Clavulanic acid 625 mg three times daily at the time of discharge. He was given outpatient follow-up appointments with a thoracic surgeon, a neurologist for nerve conduction studies (NCS) and infectious disease consultant. He was followed for a further six months and his NCS was normal. His neurological deficit did not recur and he remained stable throughout the follow-up period after discharge.

## Discussion

Chest infections or pneumonia due to bacterial infections can present as para-pneumonic effusions, complicated parapneumonic effusions, and empyema [1]. Empyema is defined as frank pus in the pleural cavity which is commonly associated with immunocompromised states as in lung cancer, diabetes mellitus, malignancy, and tuberculosis [2-4,8]. A rare complication of empyema is empyema necessitasis (EN) or empyema necessitans (EN), which develops due to retention of infected pleural fluid leading to fistula formation from the pleural cavity to the chest wall resulting in asymmetry and a prominent swelling over it [8]. EN typically develops in over four to eight weeks and is associated with pain, swelling, and lymphadenopathy of the anterolateral chest, often without fever [9]. The most common site of involvement is in the anterior chest wall, specifically, midclavicular and anterior axillary line and between the second and sixth intercostal spaces. Moreover, it is also reported to involve bronchus, vertebral column, diaphragm, breast, mediastinum, retroperitoneum, esophagus, pericardium, flank, or groin [10].

There are reviewed articles by Sindel (1940), Freeman et al. (2004) and Llamas-Velasco et al. (2010), respectively, who described mycobacterium tuberculosis as the most common cause of EN cases. They also highlighted the importance of antibiotics in its management due to the decline in the mortality rate from 66% in the pre-antibiotic era to 5% nowadays [10]. In literature, most reported cases described the association of EN with bacterial pathogens like Mycobacterium, Staphylococcus aureus (MSSA and MRSA), and Actinomycosis [10-13]. In our case, the patient initially was treated for empyema due to ESBL E. coli organism with appropriate antibiotics and chest tube thoracostomy for drainage of the infected fluid. But after three weeks, he presented again with pleuritic chest pain and new acute onset of left arm weakness which was diagnosed as EN with brachial plexus impingement after thorough investigation. This time the culture of the fluid grew Prevotella oris. One of the retrospective review studies of empyema cases caused by anaerobic bacteria has described Prevotella species as the most common isolate [5]. But EN itself has not been reported much in the literature in association with Prevotella oris leading to brachial plexopathy thus making it an unusual and challenging case [14].

## Conclusions

We want to emphasize the importance of prompt management and follow-up in such a challenging and rare presentation of EN. In such cases, the aim is early identification of the organism, initiation of antibiotics with drainage of infected pleural effusion as soon as possible to avoid any fatal consequences. Controlling underlying etiology like diabetes mellitus, as in our case, is also important for a healthy quality life and prevention of further complications.
